# Assessment of myocardial salvage in patients with STEMI undergoing thrombolysis: ticagrelor versus clopidogrel

**DOI:** 10.1186/s12872-022-02735-1

**Published:** 2022-07-02

**Authors:** Stylianos Petousis, Michalis Hamilos, Konstantinos Pagonidis, Panos Vardas, Georgios Lazopoulos, Ioannis Anastasiou, Evangelos Zacharis, George Kochiadakis, Emmanouil Skalidis

**Affiliations:** 1 Cardiology Department, University Hospital of Heraklion, Voutes and Stavrakia, 71110 Heraklion, Crete Greece; 2 School of Medicine, University of Crete, Heraklion, Greece; 3 Hellenic Cardiovascular Research Society, Athens, Greece; 4 Division of Cardiac Surgery, University Hospital of Heraklion, Heraklion, Greece

**Keywords:** STEMI, Thrombolysis, Clopidogrel, Ticagrelor, Myocardial salvage

## Abstract

**Background:**

In the setting of ST-segment elevation myocardial infarction (STEMI), the faster and stronger antiplatelet action of ticagrelor compared to clopidogrel, as well as its pleiotropic effects, could result in a greater degree of cardioprotection and final infarct size (FIS) limitation. The aim of our study was to comparatively evaluate the effect of ticagrelor and clopidogrel on myocardial salvage index (MSI) in STEMI patients undergoing thrombolysis.

**Methods:**

Forty-two STEMI patients treated with thrombolysis were randomized to receive clopidogrel (n = 21) or ticagrelor (n = 21), along with aspirin. Myocardial area at risk (AAR) was calculated according to the BARI and the APPROACH jeopardy scores. FIS was quantified by cardiac magnetic resonance imaging (CMR) performed 5–6 months post-randomization. MSI was calculated as (AAR-FIS)/AAR × 100%. Primary endpoint of our study was MSI. Secondary endpoints were FIS and CMR-derived left ventricular ejection fraction (LVEF) at 5 –6 months post-randomization.

**Results:**

By using the BARI score for AAR calculation, mean MSI was 52.25 ± 30.5 for the clopidogrel group and 54.29 ± 31.08 for the ticagrelor group (*p* = 0.83), while mean MSI using the APPROACH score was calculated at 51.94 ± 30 and 53.09 ± 32.39 (*p* = 0.9), respectively. Median CMR-derived FIS—as a percentage of LV—was 10.7% ± 8.25 in the clopidogrel group and 12.09% ± 8.72 in the ticagrelor group (*p* = 0.6). Mean LVEF at 5–6 months post-randomization did not differ significantly between randomization groups.

**Conclusions:**

Our results suggest that the administration of ticagrelor in STEMI patients undergoing thrombolysis offer a similar degree of myocardial salvage, compared to clopidogrel.

**Supplementary Information:**

The online version contains supplementary material available at 10.1186/s12872-022-02735-1.

## Introduction

Primary percutaneous coronary intervention (PCI) represents the preferred modality of reperfusion in the setting of ST-segment elevation myocardial infarction (STEMI), when it can be performed within the recommended time limits and by competent teams [1]. Under these terms, primary PCI results in better clinical outcomes compared to thrombolysis; however, in cases where these conditions are not met, thrombolysis is indicated as the reperfusion modality of choice, in the absence of contraindications [1]. Regardless of reperfusion modality, dual antiplatelet therapy (DAPT)—consisting of low-dose aspirin and a P2Y_12_-receptor inhibitor- is recommended.

While the more potent antiplatelet agent ticagrelor has a proven clinical benefit compared to clopidogrel in patients with STEMI undergoing primary PCI and is recommended as the P2Y_12_-inhibitor of choice in this context [2], its use in combination with a fibrinolytic agent has not been sufficiently studied in patients with STEMI treated with thrombolysis and is discouraged because of bleeding risk concerns [1]. Bleeding events are increased in patients with acute coronary syndromes treated invasively, particularly in those with STEMI [3] and a decrease of hemoglobin values of ≥ 3 g/dl, even if it is not apparent, is associated with an increased risk of all-cause mortality at one year [4].

On the other hand, clopidogrel’s antiplatelet action requires successive, time-consuming metabolic steps, a fact that results in a delayed achievement of platelet inhibition [5]. Moreover, a not negligible proportion of patients demonstrate a genetically determined resistance to clopidogrel, and the level of responsiveness to the agent can be unpredictable, to a certain degree [5]. Ticagrelor conversely, is characterized by a significantly more potent, predictable and faster platelet inhibition, overcoming these main limitations of clopidogrel. As platelet reactivity is substantially increased in the setting of acute myocardial infarction and is even more pronounced after thrombolysis [6, 7] these pharmacokinetic and pharmacodynamic advantages of ticagrelor might be particularly important in this setting. Additionally, several studies suggest that treatment with ticagrelor results in increased tissue levels of adenosine [8–11] and it seems that is also involved in other important and multiple cardioprotective pathways [12, 13]. These actions could be theoretically translated into a greater degree of preservation of coronary microcirculation, and ultimately cardioprotection. Coronary microvascular dysfunction can be detected in a significant proportion of reperfused patients with STEMI and it has been associated with larger infarct size, poor recovery of left ventricular (LV) systolic function and worse clinical prognosis [14].

The aim of our study was to investigate the potential advantageous effects of ticagrelor compared to clopidogrel in terms of myocardial salvage in patients with STEMI treated with thrombolysis, by utilizing in a combining way late gadolinium enhancement (LGE) cardiovascular magnetic resonance imaging (CMR) and well-established angiographic scores (the BARI and the APPROACH angiographic scores).

## Material and methods

### Study population

Our study included a subpopulation of patients of the MIRTOS study (ClinicalTrials.gov Identifier: NCT02429271) [15], a prospective, randomized open-label multicenter clinical trial that took place in Greece between October 2015 and July 2018. Inclusion and exclusion criteria were the same as for the principal study. Additional exclusion criteria were a history of myocardial infarction in the same territory of the culprit coronary artery and a contraindication to CMR. The study, which had been approved by Institutional Review Board of the University Hospital of Heraklion, was conducted in accordance to the declaration of Helsinki and informed consent was obtained from all subjects.

We aimed to include 42 patients with STEMI initially presenting to community hospitals, in which thrombolysis was the reperfusion modality of choice per current guidelines on grounds of non-feasibility of primary PCI within the time limit of 120 min from first medical contact. These patients had been previously randomized in a 1:1 ratio to treatment with clopidogrel or ticagrelor along with low-dose aspirin. Thrombolysis method took place as follows: In all subjects a orally loading dose of aspirin of 150–300 mg o was given routinely (maintenance dose of 75–100 mg orally od thereafter). If a subject had already taken aspirin within 12 h prior to presentation, aspirin was started next day. For subjects assigned to Ticagrelor a single 180 mg loading dose was given (maintenance dose of 90 mg orally bid thereafter). For patients assigned to Clopidogrel a loading dose of 300 mg was given (maintenance dose of 75 mg od thereafter). A weight adjusted dose of Enoxaparin or Unfractioned Heparin, among with a weight adjusted i.v. fibrin specific agent was used (tenecteplace in our substudy) as part of the antithrombotic regimen. The aim in community hospitals was to initiate thrombolysis within 30 min. Criteria for successful thrombolysis were: ST-segment resolution of more than 50% at 60–90 min, typical reperfusion arrhythmia, and disappearance of chest pain.

Other medications -including anticoagulants, b-blockers, statins, angiotensin converting enzyme inhibitors (ACE-inhibitors) nitrates, calcium channel blockers- were thereafter administered as appropriate according to treating physicians’ judgment. Patients were subsequently transferred to our PCI-capable center for coronary angiography within 72 hours. In cases where thrombolysis was unsuccessful, patients were immediately transferred to our center for rescue PCI.

After coronary angiography, PCI was performed, when indicated, with implantation of drug-eluting stents. During intervention, the performance of dilations and thrombus aspiration, as well as the administration of glycoprotein IIb/IIIa-receptor inhibitors and additional doses of anticoagulants took place according to the operator’s judgment.

Subjects were comprehensively evaluated at their discharge from hospital. Per protocol of the main study (MIRTOS STUDY) all study participants underwent scheduled visits at 30 and 90 days post-randomization, whereby comprehensive clinical and laboratory evaluation, as well as medication review (including statins) and adherence assessment, were performed. The aim of statin therapy was to archieve a LDL-C reduction by > 50% and/or to archieve LDL-C values of < 70 mg/dL according to the guidelines at that time. If LDL-C values were not archieved after 4–6 weeks with maximally tolerated dose of statins, ezetimibe was added.

**Fig. 1:**
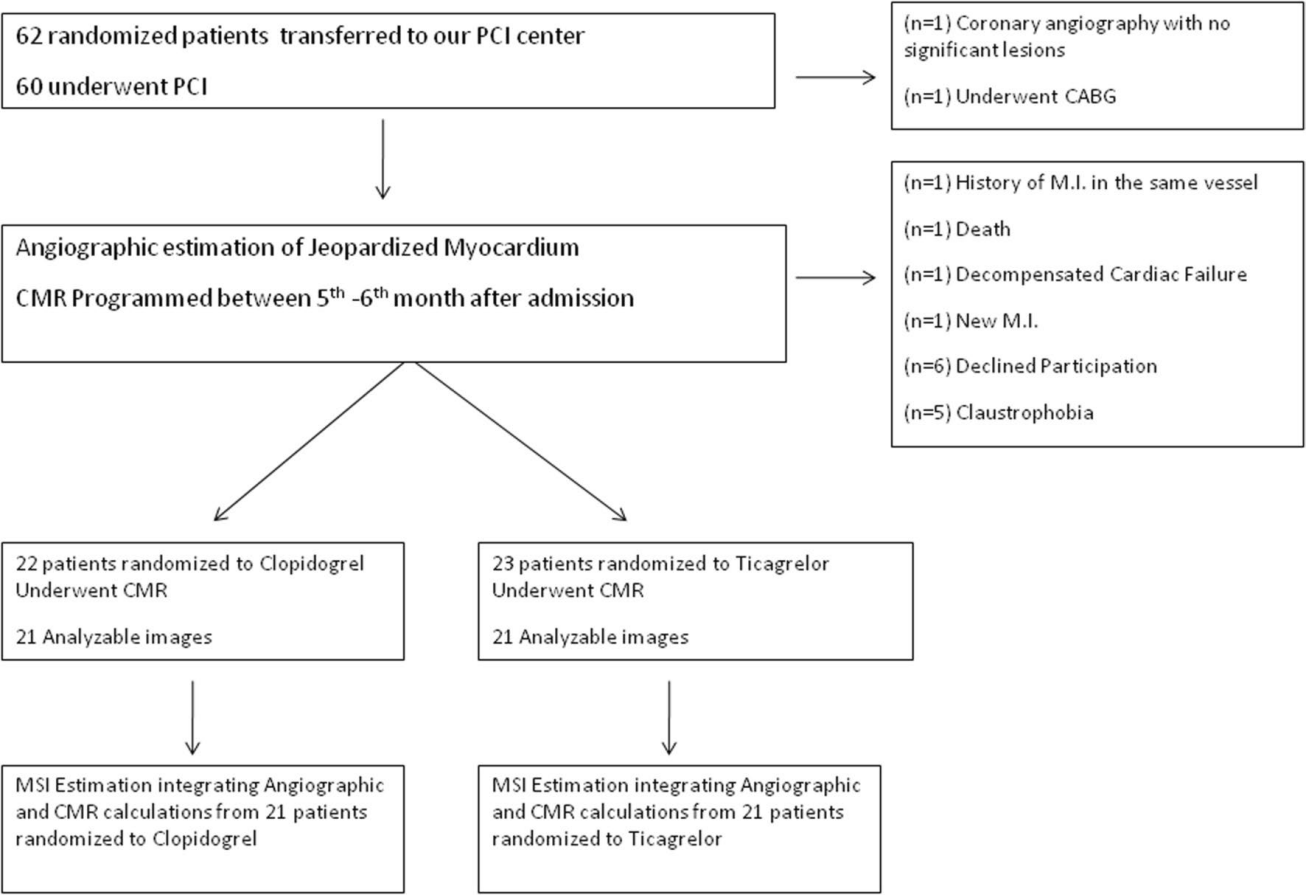
Study flow chart. *PCI* Percutaneous coronary intervention, *MI* Myocardial infarction, *CABG* Coronary artery bypass grafting, *CMR* Cardiac magnetic resonance imaging, *MSI* Myocardial Salvage Index

From 62 initially enrolled randomized patients, 20 were excluded for various reasons and our final study population consisted of 42 patients (Fig. 1), 21 of which received clopidogrel and 21 ticagrelor. These 62 patients were enrolled *exclusively* in our center (University Hospital of Heraklion, Cardiology Department, Greece) and the participation in the CMR study was requested from the patients on their arrival from secondary hospitals of our region.

### Measurements

#### Angiographic analysis

In all patients who underwent PCI, the initial myocardial area at risk was estimated according to the Bypass Angioplasty Revascularization Investigation Myocardial Jeopardy Index (BARI) and Alberta Provincial Project for Outcome Assessment in Coronary Heart Disease (APPROACH) angiographic scores from two interventional cardiologists in our center in a blinded manner, based on the methodologies described below. In the BARI scoring system, each terminal branch of the coronary arteries (terminal part of the left anterior descending artery, diagonals, septal perforators, obtuse marginal branches of the left circumflex artery, ramus intermedius, posterior descending and posterolateral branches of the right coronary artery) is graded from 0 to 3 according to its size, by standardized criteria. Subsequently, the sum of the grades assigned to terminal branches of the infarct-related artery distal to the culprit lesion is calculated and divided by the sum of the grades of all terminal branches (total score of the left ventricle), thereby providing myocardium at risk as a percentage of the LV [16]. The APPROACH scoring system and its modified version, on the other hand, which were established on anatomopathological human studies, take into account the anatomical point of the culprit lesion, coronary arterial dominance and the size of secondary coronary arteries for the calculation of the jeopardized portion of left ventricular myocardium [17–19].

Additionally, in each coronary angiography, pre-PCI Thrombolysis In Myocardial Infarction (TIMI) flow was assessed from cine images acquired prior to the PCI procedure. In cases of total occlusion of the culprit vessel, angiographic collateral flow was estimated according to the Rentrop classification (grade 0: absence of collateral filling; grade 1: filling of the side branches of the culprit vessel; grade 2: partial filling of the epicardial segment of the vessel through collaterals; grade 3: total filling of the epicardial segment of the vessel through collaterals).

#### Cardiac magnetic resonance imaging

We choosed to evaluate final infarct size between 5 and 6^th^ month which is the most studied follow up time interval in relevant CMR studies [20].

Analyzable CMR images were obtained from 42 randomized patients (Clopidogrel: n = 21 Ticagrelor: n = 21) at 5 to 6 months post-STEMI. A 1.5-T scanner (Magnetom Sonata, SiemensMedical Solutions) was used for all MRI examinations. The MRI protocol included a functional study of the LV using an ECG-gated breath-hold segmented steady-state free precession (SSFP; true fast imaging with steady-state free precession cine sequence (TR/TE: 2.0/1.0 ms; flip angle: 65°) with a slice thickness of 8 mm. After three standard long-axis slices were obtained, contiguous short-axis slices were acquired to cover the entire LV without an interslice gap.

After injection of 0.1 mmol/kg body weight of Gadobutrol (Gadovist, Bayer), LGE scans were obtained in three long-axis and all short-axis orientations by using a breath-hold ECG-triggered 2D inversion recovery turbo FLASH sequence (TR/TE: 8/4 ms; flip angle: 25°).

Images were acquired subsequently up to 15 min after injection. The inversion time (TI, nonselective inversion pulse) was adjusted manually between 180 and 300 ms to null the signal of normal myocardium. Depending on the field of view, the typical in-plane resolution was 1.6 × 1.3mm^2^ for all sequences.

All MRI examinations were interpreted by an experienced radiologist who was blinded for the study drug, on a workstation with dedicated software. Global LV function was quantified using Segment v3.0 R7946 (http://segment.heiberg.se). Indatasets with LV myocardial LGE after Gadobutrol injection, transmural extent of LGE (subendocardial, mid-myocardial, subepicardial, and transmural) was evaluated. The localization of LGE within the LV was described by using the American Heart Association’s segmentation of the LV. Infarct size was quantified using Segment v3.0 R7946 (http://segment.heiberg.se).

#### Myocardial salvage index (MSI)

The primary endpoint of our study was the MSI as a percentage of the LV myocardium, calculating the initial Myocardial Area at risk (AAR) both by the BARI and by the APPROACH scoring methods. Final infarct size (FIS) as percentage of LV mass was estimated by CMR. Subsequently the MSI was calculated by the formula:

$$\frac{AAR - FIS}{{AAR}} \times \, 100\% .$$

### Sample calculation and statistical analysis

Taking in consideration previous studies [21, 22] we assumed a median of MSI of about 47% for the reference group (clopidogrel) and a standard deviation of 16%. We calculated that we needed 21 patients per arm in order to detect as significant at the 5% of error risk, a difference of 14% in the MSI between the two groups (corresponding to a 30% of relative treatment effect) with 80% power. Categorical variables were compared applying the chi-square testing. Data were assessed for normal distribution using the D’Agostino—Pearson and Shapiro-Wilk normality tests (Additional file 1). Continuous variables are expressed as the mean ± SD and compared according the unpaired t-test when normally distributed, or by non parametric tests (Mann–Whitney test) for non-normally distributed values. The statistical tests we effectuated were two-sided and a value of *p* < 0.05 was considered significant. Statistical analysis and graphs were obtained using software GraphPad Prism version 7 (GraphPad Software Inc., San Diego, California, USA).

## Results

### Patients’ characteristics and therapeutic procedures time-intervals

Patients’ baseline clinical characteristics are shown in Table 1. Symptom-to-needle and needle-to-balloon time intervals, as well as pharmacologic treatments administered, did not differ significantly between the two groups. There was a trend towards better glomerular filtration rate values for the clopidogrel group (*p* = 0.072). Adherence to study medication (ticagrelor or clopidogrel) was systematically evaluated at 30 days and at 90 days post-randomization by tablet count, and was estimated at > 90% for all patients. Regarding concomitant medications—including low-dose aspirin- no major compliance issues were reported by study participants.

**Table 1:** Patients’ clinical characteristics, pharmaceutical treatment and procedure time intervals

	Clopidogrel n = 21	Ticagrelor n = 21	*p*-value
Males	17 (80.9%)	20 (95.2%)	ns
Females	4 (19%)	1 (4.7%)	ns
Age (years)	57.9 ± 7.04	53.71 ± 9.33	ns
Risk factors			
Hypertension	11 (52.3%)	9 (42.8%)	ns
Dyslipidemia	7 (33.3%)	8 (38%)	ns
Diabetes	7 (33.3%)	4 (19%)	ns
Smokers	17 (80.9%)	18 (85.7%)	ns
Laboratory parameters			
Hematocrit (%)	44.6 ± 4.28	43.54 ± 2.92	ns
Platelet count (K/μl)	276.4 ± 79.42	253 ± 81.07	ns
Estimated glomerular filtration rate (ml/min)	110.8 (62–130.8)	95 (69.95–101)	ns
Time intervals			
Symptom-to-Needle time (hours)	2.33 (2–5.2)	1.92 (1.5–4.1)	ns
Needle-to-Balloon time (hours)	33.25 ± 19.67	31.78 ± 20.27	ns
Successful thrombolysis	19/21 (90.4%)	20/21 (95.2%)	ns
Fibrinolytic agent used			
Tenecteplase	21 (100%)	21 (100%)	ns
Concomitant medications at inclusion			
Unfractionated heparin	1 (4.76%)	0 (0%)	ns
Low-molecular-weight heparin	20 (95.2%)	21 (100%)	ns
Aspirin	21 (100%)	21 (100%)	ns
Statin	6 (28.57%)	5 (23.8%)	ns
Beta-blocker	4 (19%)	3 (14.3%)	ns
Nitroglycerine	7 (33.3%)	6 (28.5%)	ns
Angiotensin converting enzyme/angiotensin receptor inhibitors	5 (23.8%)	7 (33.3%)	ns
Medication at discharge from hospital			
Aspirin	21 (100%)	21 (100%)	ns
High dose Statin	21 (100%)	21 (100%)	ns
Atorvastatin 40 or 80 mg	16 (76.2%)	14 (66.6%)	ns
Rosuvastatin 20 or 40 mg	5 (23.8%)	7 (33.3%)	ns
Beta-blockers	16 (76%)	17 (80%)	ns
Angiotensin converting enzyme/angiotensin receptor inhibitors	18 (85.7%)	16 (76%)	ns
Mineralocorticoid receptor antagonists	2 (9.5%)	3 (14.2%)	ns

Data are presented as mean ± Standard Deviation, median (quartiles 1–3), or number and percentage of patients. *p*-values express comparison between the two groups (Clopidogrel versus Ticagrelor)

### Angiographic and PCI-related parameters

Coronary artery disease extent was similar between the two randomization groups. No significant differences were detected regarding pre-PCI TIMI flow values. Data regarding coronary angiography and PCI procedure are presented in Table 2.

**Table 2:** Coronary angiography and PCI-procedure data

	Clopidogrel n = 21	Ticagrelor n = 21	*p*-value
Culprit lesion location			
LAD	5 (23.8%)	6 (28.5%)	ns
RCA	12 (57.1%)	12 (57.1%)	ns
LCX	4 (19%)	3 (14.3%)	ns
Disease extent			
1VD	12(57.1%)	13 (61.9%)	ns
2VD	7 (33.3%)	5 (23.8%)	ns
3VD	2 (9.5%)	3 (14.3%)	ns
TIMI pre-PCI			
TIMI 3	17 (80.9%)	18 (85.7%)	ns
TIMI 2	2 (9.5%)	1 (4.7%)	ns
TIMI 1	0 (0%)	1 (4.7%)	ns
TIMI 0	2 (9.5%)	1 (4.7%)	ns
Rentrop II–III collaterals	2 (9.5%)	1 (4.7%)	ns
PCI procedure			
Number of balloons used	0.95 ± 0.86	0.76 ± 0.88	ns
Inflation pressure (atm)	14.86 ± 3.2	15.33 ± 2.39	ns
Number of stents implanted	1.43 ± 0.5	1.476 ± 0.68	ns
Total stent length (mm)	30.19 ± 14.63	28.67 ± 15.48	ns
Stent diameter (mm)	3.23 ± 0.49	2.25 ± 0.44	ns
Thrombus aspiration performed	1 (4.7%)	0 (0%)	ns
Area at Risk (%)			
Area at risk BARI (%)	22.76 ± 7.33	25.84 ± 5.92	0.14
Area at risk APPROACH (%)	22.35 ± 6.9	25.16 ± 5.7	0.15

Data are presented as mean ± Standard Deviation, median (quartiles 1–3), or number and percentage of patients. *p*-values express comparison between the two groups (Clopidogrel versus Ticagrelor)

*LAD* Left anterior descending artery, *RCA* Right coronary artery, *LCX* Left circumflex artery, *VD* Vessel disease, *TIMI* Thrombolysis In Myocardial Infarction flow grade, *PCI* Percutaneous coronary intervention, Area at risk—BARI/APPROACH (%): Myocardial area at risk calculated by using the BARI/APPROACH scoring systems (% of left ventricular myocardium)

Angiographically calculated AAR (jeopardized myocardium) was comparable between the clopidogrel and ticagrelor groups, as assessed by the BARI score 22.76 ± 7.33 versus 25.84 ± 5.92 (*p* = 0.14) and the APPROACH score 22.35 ± 6.9 versus 25.16 ± 5.7 (*p* = 0.15), respectively.

### CMR-derived measurements

FIS, measured as percentage of total LV volume or as absolute value of myocardial volume, did not differ significantly between the two groups. In addition, no differences were noted in terms of maximum or mean transmurality of the myocardial infarct. Left ventricle ejection fraction (LVEF), stroke volume index (SVI), end-systolic and end-diastolic LV volumes were also comparable between randomization groups, although a trend towards higher SVI values was observed for the ticagrelor group (*p* = 0.079). Primary CMR-derived variables are summarized in Table 3.

**Table 3:** CMR-derived measurements and Myocardial Salvage Index

CMR-derived measurements (5–6 month post-randomization)	Clopidogrel (n = 21)	Ticagrelor (n = 21)	*p*-value
Timing of CMR from Thrombolysis (Months)	5.66 ± 0.69	5.48 ± 0.59	0.38
EDVI (ml/m^2^)	69.28 ± 24.36	71.48 ± 13.69	0.72
ESVI (ml/m^2^)	34.83 ± 20.46	33.85 ± 11.66	0.85
SV (ml)	70.39 ± 18.99	79.71 ± 19.21	0.12
SVI (ml/m^2^)	35.28 (28.51–38.6)	39.04 (31.05–45.23)	0.079
LVEF (%)	51.94 ± 12.18	54.14 ± 10.37	0.53
Infarct total extent (% LV)	24.4 ± 15.66	26.65 ± 17.49	0.66
Maximal transmurality (%)	86.48 (89–100)	82.38 (71–100)	0.87
Mean transmurality (%)	48.1 ± 22.2	45.13 ± 20.66	0.65
Scar Volume (ml)	9.96 (4.84–26.97)	17.27 (4.02–24.14)	0.49
Final infarct Size (% LV)	10.7 ± 8.25	12.09 ± 8.72	0.6
MSI-BARI	52.25 ± 30.5	54.29 ± 31.08	0.83
MSI-APPROACH	51.94 ± 30.01	53.09 ± 32.39	0.9

Data are presented as mean ± Standard Deviation, median (quartiles 1–3), or number and percentage of patients. *p*-values express comparison between the two groups (Clopidogrel versus Ticagrelor)

*CMR* Cardiac magnetic resonance imaging, *EDVI* Left ventricular end-diastolic volume index, *ESVI* Left ventricular end-systolic volume index, *SV* Stroke volume, *SVI* Stroke volume index, *LV* Left ventricle, *LVEF* Left ventricular ejection fraction, *MSI-BARI/APPROACH* Myocardial Salvage Index calculated after estimation of Area At Risk with the use of the BARI/APPROACH angiographic scoring systems, % LV Percentage of left ventricular myocardium

### Primary endpoint

**Fig. 2:**
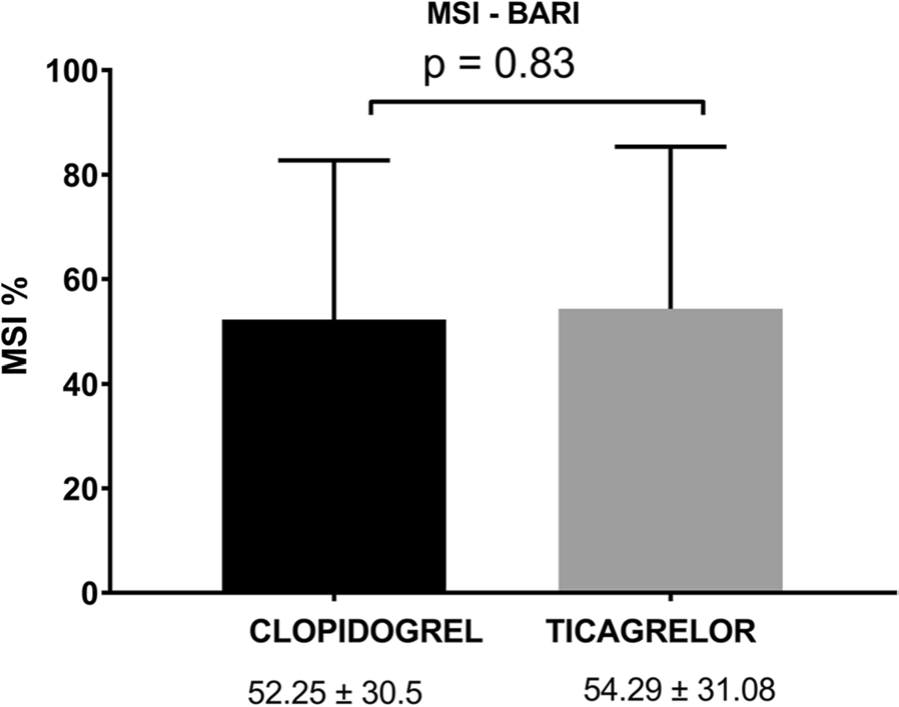
Primary endpoint analysis: Myocardial Salvage Index after calculation of Area At Risk by the BARI angiographic score. *MSI (%)* Myocardial Salvage Index as a percentage of left ventricular myocardium

**Fig. 3:**
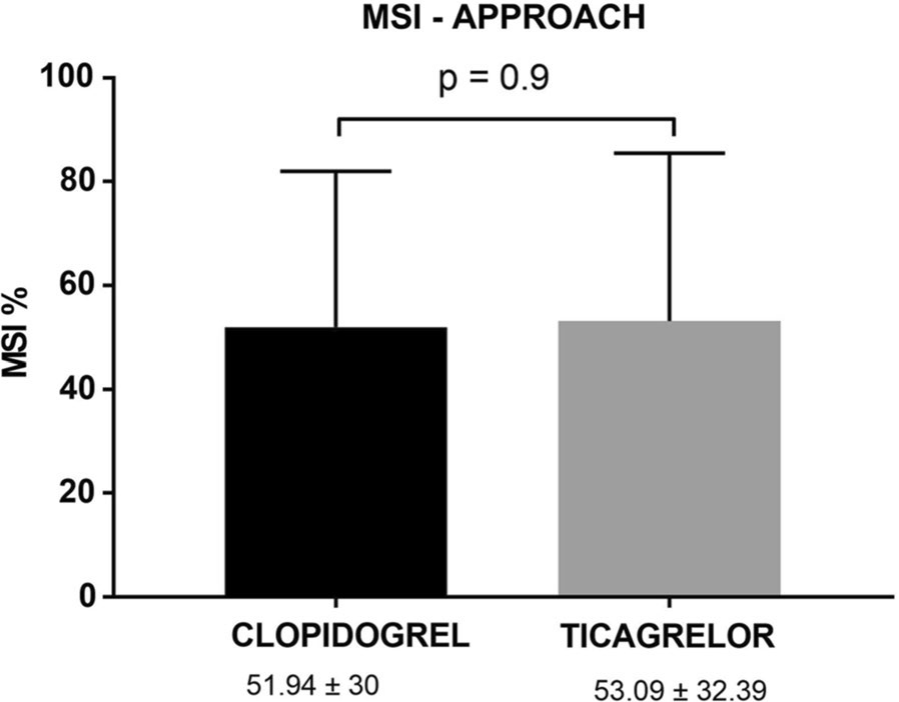
Primary endpoint analysis: Myocardial Salvage Index after calculation of Area At Risk by the APPROACH angiographic score. *MSI (%)* Myocardial Salvage Index as a percentage of left ventricular myocardium

Our study’s primary endpoint, the MSI, combining angiographic and CMR calculations, did not differ significantly between the two groups, whether estimating the initial percentage of the jeopardized myocardium according the BARI or the APPROACH scoring system (Figs. 2, 3, respectively).

## Discussion

In the setting of acute myocardial infarction, a more robust and earlier-onset platelet inhibition could result in a smaller FIS and a greater degree of myocardial salvage. Earlier studies suggest that a higher loading dose of clopidogrel (600 mg rather than 300 mg), limits myocardial infarct size more effectively in patients with STEMI undergoing primary PCI [21, 23]. In addition, there is evidence that the newer P2Y_12_-inhibitors ticagrelor and prasugrel are more effective in this context, although it must be emphasized that there is no uniformity of the results about the degree of myocardial infarction mitigation [24–27]. Although primary PCI is the indicated modality of reperfusion in the setting of STEMI, when it cannot be performed within the recommended time limits and by adequately trained teams, thrombolysis is the reperfusion treatment of choice. A significant proportion of patients undergo this treatment option in countries where a primary PCI network is poorly developed or geography poses obstacles to timely patient transfer to PCI centers, as is the case in Greece [28]. We aimed to investigate if this group of patients could benefit from a newer, more efficacious antiplatelet agent. We thus sought to investigate whether the more efficient antiplatelet action of ticagrelor, along with its other reported cardioprotective properties [12, 13]—as compared to clopidogrel- could translate into a greater degree of myocardial preservation in STEMI patients undergoing thrombolysis, a treatment that can per se increase platelet reactivity. The robust action of ticagrelor could potentially attenuate the detrimental effects of this intense platelet activation by decreasing further thrombus formation and distal embolization, by diminishing local vasospasm due to vasoconstricting substances—such as serotonin and endothelin- released from activated platelets, as well as by attenuating the process of inflammation. Additionally, a loading dose of 300 mg of clopidogrel requires a minimum of 6 h to achieve a sufficient level of platelet inhibition, while the full-scale antiplatelet effect of a 180 mg loading dose of ticagrelor can be evident within 2 h from administration. Moreover, given that adenosine improves coronary microcirculation, while also possessing a well-recognized role in platelet inhibition and in the attenuation of inflammatory processes, the suggested—albeit questioned by some researchers [29, 30] -adenosine-mediated pleiotropic effects of ticagrelor could also be of importance in this setting [31–33].

In line with these data, it has been shown that treatment with ticagrelor can significantly ameliorate microvascular injury in patients with STEMI undergoing primary PCI, reducing the index of microvascular resistance (IMR) by 35% [27]. Accordingly, another study reported that ticagrelor can effectively reduce the size of myocardial infarct in patients with STEMI undergoing primary PCI, compared to clopidogrel, as shown by CMR [34]. However, the beneficial actions of this agent have not been studied yet in terms of myocardial salvage or FIS in patients reperfused with thrombolysis.

Despite its small sample size, our study is the first that approaches, through CMR analysis and myocardial salvage calculations, the question of myocardial infarct mitigation in patients with STEMI undergoing thrombolysis, while receiving two different generations of antiplatelet agents.

Endpoints (MSI and FIS) were defined based on their well-recognized prognostic significance in STEMI patients and their utility as reliable indicators of efficacy of reperfusion therapy [35–37].

Our study indicates that, in patients undergoing thrombolysis, the use of either P2Y_12_-inhibitor on top of low-dose aspirin, is accompanied by a comparable degree of myocardial salvage, infarct size and LVEF at 5 to 6 months post-STEMI. Our investigation was not adequately powered to detect significant differences in FIS values between the two groups, but it is the first study to investigate this parameter under this clinical context. On the other hand, we took into consideration the initial AAR while calculating MSI, thus minimizing the effect of discrepancies in the extent of jeopardized myocardium between the two groups.

The similar observed effectiveness of both agents suggests that the pharmacokinetic and pharmacodynamic advantages of ticagrelor over clopidogrel, cannot overcome the thrombolysis-induced platelet overreactivity. Added to that, the effect of other parameters, such as the total ischemic time—which can be longer in patients initially reperfused with thrombolysis rather than primary PCI- are of paramount importance in determining the extent of myocardial infarction. The observed equivalence of the cardioprotective effect of ticagrelor and clopidogrel in the context of lytic therapy is consistent with the findings of the main MIRTOS study, where no significant differences in microvascular injury, estimated by the post-PCI corrected TIMI frame count values, were found between the two randomization groups [15]. It should also be mentioned that the dispersion of MSI values in our analysis was greater than we initially expected, resulting in larger values of standard deviation. However, our study had a pilot role and larger future trials are needed for our findings to be confirmed.

There were some limitations regarding our research. First, it was a small, single center study. However it is the first work to investigate the size of infarct size and the myocardial salvage of randomized patients with STEMI who underwent thrombolysis, previously randomized to a ticagrelor or a clopidogrel medication. Second, while it is known that CMR represents a widely used method of measuring the myocardial AAR during the acute phase of myocardial infarction, in our study this parameter was estimated using angiographic scoring systems. However, it should be emphasized that AAR calculated by either the BARI or the APPROACH score demonstrates a very good correlation with the respective CMR-derived values, particularly in patients presenting with STEMI [38, 39]. In fact, estimation of MSI by combining angiographic assessment of AAR and FIS estimation by CMR has been outlined as a valid alternative [19]. Thus, this approach allowed us to overcome the difficulty posed by the limited availability-till recently- of CMR in our hospital. Moreover, CMR can overestimate the initial area at risk of myocardium during the acute phase of myocardial infarction, because of the presence of myocardial edema and there are also several concerns about the optimal timing of the examination [40]. Third, a significant number of patients did not undergo coronary angiography within 24 h, as current guidelines recommend, something that we however expected, considering “real life” practices in our region.

## Conclusions

Our study suggests that ticagrelor does not improve myocardial salvage index compared to clopidogrel in STEMI patients who underwent thrombolysis, and therefore the cardioprotective effect of each agent in this setting appears to be similar. Nevertheless, our sample size was limited and larger studies are needed henceforth for more definite conclusions to be drawn.

## Supplementary Information

**Additional file1: Table S1.** D’Agostino and Shapiro-Wilk tests for Cardiac Magnetic Resonance Timing. **Table S2.** D’Agostino and Shapiro-Wilk tests for Area at risk calculated according the APPROACH JEOPARDY SCORE. **Table S3.** D’Agostino and Shapiro-Wilk tests for Area at risk calculated according the BARI JEOPARDY SCORE. **Table S4.** D’Agostino and Shapiro-Wilk tests for infarct size as percentage of Left Ventricle (LV) estimated by Cardiac Magnetic Resonance (CMRI). **Table S5.** D’Agostino and Shapiro-Wilk tests for Myocardial Salvage Index (MSI) according APPROACH JEOPARDY SCORE. **Table S6.** D’Agostino and Shapiro-Wilk tests for Myocardial Salvage Index (MSI) according the BARI JEOPARDY SCORE. **Table S7.** D’Agostino and Shapiro-Wilk tests for Left Ventricle’s Ejection Fraction estimated by Cardiac Magnetic Resonance (CMRI). **Table S8.** D’Agostino and Shapiro-Wilk tests for Left Ventricle’s Stroke Volume in ml estimated by Cardiac Magnetic Resonance (CMRI). **Table S9.** D’Agostino and Shapiro-Wilk tests for Left Ventricle end systolic volume index (ml/m2). **Table S10.** D’Agostino and Shapiro-Wilk tests for Left Ventricle End Diastolic Volume Index (ml/m2). **Table S11.** D’Agostino and Shapiro-Wilk tests for myocardial infarct mean transmurality Values as percentage of Left Ventricle.

## Data Availability

The datasets generated and/or analysed during the current study are not publicly available due to the lack of dedicated and approved for this purpose repositories at our site, but are available from the corresponding author on reasonable request.
