# Supplementary material for: Assessment of myocardial salvage in patients with STEMI undergoing thrombolysis: ticagrelor versus clopidogrel
Source: BMC Cardiovasc Disord. 2022 Jul 2;22:301. doi: 10.1186/s12872-022-02735-1 (PMC9250208; doi:10.1186/s12872-022-02735-1)
Supplement: Supplementary file 1 — Additional file1: Table S1. D’Agostino and Shapiro-Wilk tests for Cardiac Magnetic Resonance Timing. Table S2. D’Agostino and Shapiro-Wilk tests for Area at risk calculated according the APPROACH JEOPARDY SCORE. Table S3. D’Agostino and Shapiro-Wilk tests for Area at risk calculated according the BARI JEOPARDY SCORE. Table S4. D’Agostino and Shapiro-Wilk tests for infarct size as percentage of Left Ventricle (LV) estimated by Cardiac Magnetic Resonance (CMRI). Table S5. D’Agostino and Shapiro-Wilk tests for Myocardial Salvage Index (MSI) according APPROACH JEOPARDY SCORE. Table S6. D’Agostino and Shapiro-Wilk tests for Myocardial Salvage Index (MSI) according the BARI JEOPARDY SCORE. Table S7. D’Agostino and Shapiro-Wilk tests for Left Ventricle’s Ejection Fraction estimated by Cardiac Magnetic Resonance (CMRI). Table S8. D’Agostino and Shapiro-Wilk tests for Left Ventricle’s Stroke Volume in ml estimated by Cardiac Magnetic Resonance (CMRI). Table S9. D’Agostino and Shapiro-Wilk tests for Left Ventricle end systolic volume index (ml/m2). Table S10. D’Agostino and Shapiro-Wilk tests for Left Ventricle End Diastolic Volume Index (ml/m2). Table S11. D’Agostino and Shapiro-Wilk tests for myocardial infarct mean transmurality Values as percentage of Left Ventricle. [file 12872_2022_2735_MOESM1_ESM.docx]

**SUPPLEMENTARY MATERIAL - STATISTICAL NORMALITY TESTS**

**Table 1:** **D’Agostino and Shapiro-Wilk tests for Cardiac Magnetic Resonance Timing**

CMRI timing in Months CLOPIDOGREL TICAGRELOR

| Number of values | 21 | 21 |
| --- | --- | --- |
|  |  |  |
| Minimum | 4,5 | 4,2 |
| 25% Percentile | 5,05 | 5,2 |
| Median | 5,6 | 5,4 |
| 75% Percentile | 6,4 | 6 |
| Maximum | 6,6 | 6,5 |
|  |  |  |
| Mean | 5,666 | 5,488 |
| Std. Deviation | 0,6945 | 0,5988 |
| Std. Error of Mean | 0,1515 | 0,1307 |
|  |  |  |
| Lower 95% CI of mean | 5,35 | 5,216 |
| Upper 95% CI of mean | 5,982 | 5,761 |
|  |  |  |
| Sum | 119 | 115,3 |
|  |  |  |
| D'Agostino & Pearson normality test |  |  |
| K2 | 4,426 | 0,07489 |
| P value | 0,1094 | 0,9632 |
| Passed normality test (alpha=0.05)? | Yes | Yes |
| P value summary | ns | ns |
|  |  |  |
| Shapiro-Wilk normality test |  |  |
| W | 0,9163 | 0,9613 |
| P value | 0,0733 | 0,5433 |
| Passed normality test (alpha=0.05)? | Yes | Yes |
| P value summary | ns | ns |

**Table 2: D’Agostino and Shapiro-Wilk tests for Area at risk calculated according the APPROACH JEOPARDY SCORE**

% AREA AT RISK APPROACH SCORE CLOPIDOGREL TICAGRELOR

| Number of values | 21 | 21 |
| --- | --- | --- |
|  |  |  |
| Minimum | 9,75 | 15,25 |
| 25% Percentile | 15,25 | 21,25 |
| Median | 24,5 | 27,5 |
| 75% Percentile | 27,75 | 27,75 |
| Maximum | 32,5 | 38,5 |
|  |  |  |
| Mean | 22,35 | 25,16 |
| Std. Deviation | 6,916 | 5,702 |
| Std. Error of Mean | 1,509 | 1,244 |
|  |  |  |
| Lower 95% CI of mean | 19,2 | 22,57 |
| Upper 95% CI of mean | 25,5 | 27,76 |
|  |  |  |
| Sum | 469,3 | 528,5 |
|  |  |  |
| D'Agostino & Pearson normality test |  |  |
| K2 | 3,579 | 0,1936 |
| P value | 0,1670 | 0,9077 |
| Passed normality test (alpha=0.05)? | Yes | Yes |
| P value summary | ns | ns |
|  |  |  |
| Shapiro-Wilk normality test |  |  |
| W | 0,8944 | 0,9316 |
| P value | 0,0273 | 0,1485 |
| Passed normality test (alpha=0.05)? | No | Yes |
| P value summary | * | ns |

**Table 3: D’Agostino and Shapiro-Wilk tests for Area at risk calculated according the BARI JEOPARDY SCORE**

% AREA AT RISK ACCORDING BARI CLOPIDOGREL TICAGRELOR

| Number of values | 21 | 21 |
| --- | --- | --- |
|  |  |  |
| Minimum | 10 | 16,67 |
| 25% Percentile | 14,64 | 20,53 |
| Median | 26 | 27,77 |
| 75% Percentile | 27,64 | 29 |
| Maximum | 35,2 | 40 |
|  |  |  |
| Mean | 22,76 | 25,84 |
| Std. Deviation | 7,332 | 5,922 |
| Std. Error of Mean | 1,6 | 1,292 |
|  |  |  |
| Lower 95% CI of mean | 19,43 | 23,14 |
| Upper 95% CI of mean | 26,1 | 28,53 |
|  |  |  |
| Sum | 478,1 | 542,6 |
|  |  |  |
| D'Agostino & Pearson normality test |  |  |
| K2 | 2,632 | 0,6586 |
| P value | 0,2682 | 0,7194 |
| Passed normality test (alpha=0.05)? | Yes | Yes |
| P value summary | ns | ns |
|  |  |  |
| Shapiro-Wilk normality test |  |  |
| W | 0,921 | 0,958 |
| P value | 0,0908 | 0,4771 |
| Passed normality test (alpha=0.05)? | Yes | Yes |
| P value summary | ns | ns |

**Table 4: D’Agostino and Shapiro-Wilk tests for infarct size as percentage of**

**Left Ventricle (LV) estimated by Cardiac Magnetic Resonance (CMRI)**

LV % of Infarct Size CLOPIDOGREL TICAGRELOR

| Number of values | 21 | 21 |
| --- | --- | --- |
|  |  |  |
| Minimum | 0 | 0 |
| 25% Percentile | 5,4 | 3,245 |
| Median | 9,35 | 13,03 |
| 75% Percentile | 15,64 | 15,97 |
| Maximum | 34 | 29,12 |
|  |  |  |
| Mean | 10,7 | 12,09 |
| Std. Deviation | 8,256 | 8,723 |
| Std. Error of Mean | 1,802 | 1,903 |
|  |  |  |
| Lower 95% CI of mean | 6,939 | 8,115 |
| Upper 95% CI of mean | 14,45 | 16,06 |
|  |  |  |
| Sum | 224,6 | 253,8 |
|  |  |  |
| D'Agostino & Pearson normality test |  |  |
| K2 | 7,153 | 0,3732 |
| P value | 0,0280 | 0,8298 |
| Passed normality test (alpha=0.05)? | No | Yes |
| P value summary | * | ns |
|  |  |  |
| Shapiro-Wilk normality test |  |  |
| W | 0,9219 | 0,9303 |
| P value | 0,0948 | 0,1395 |
| Passed normality test (alpha=0.05)? | Yes | Yes |
| P value summary | ns | ns |
|  |  |  |

**Table 5:** **D’Agostino and Shapiro-Wilk tests for Myocardial Salvage Index (MSI) according APPROACH JEOPARDY SCORE**

MSI ACCORDING APPROACH SCORE CLOPIDOGREL TICAGRELOR

| Number of values | 21 | 21 |
| --- | --- | --- |
|  |  |  |
| Minimum | 0 | 0 |
| 25% Percentile | 29,52 | 24,29 |
| Median | 51,84 | 50,66 |
| 75% Percentile | 74,98 | 83,43 |
| Maximum | 100 | 100 |
|  |  |  |
| Mean | 51,94 | 53,09 |
| Std. Deviation | 30,01 | 32,39 |
| Std. Error of Mean | 6,549 | 7,068 |
|  |  |  |
| Lower 95% CI of mean | 38,28 | 38,35 |
| Upper 95% CI of mean | 65,61 | 67,83 |
|  |  |  |
| Sum | 1091 | 1115 |
|  |  |  |
| D'Agostino & Pearson normality test |  |  |
| K2 | 1,101 | 1,524 |
| P value | 0,5766 | 0,4667 |
| Passed normality test (alpha=0.05)? | Yes | Yes |
| P value summary | ns | ns |
|  |  |  |
| Shapiro-Wilk normality test |  |  |
| W | 0,9656 | 0,9296 |
| P value | 0,6344 | 0,1351 |
| Passed normality test (alpha=0.05)? | Yes | Yes |
| P value summary | ns | ns |

**Table 6: D’Agostino and Shapiro-Wilk tests for Myocardial Salvage Index (MSI) according the BARI JEOPARDY SCORE**

MSI ACCORDING BARI SCORE CLOPIDOGREL TICAGRELOR

| Number of values | 21 | 21 |
| --- | --- | --- |
|  |  |  |
| Minimum | 1,1 | 6,95 |
| 25% Percentile | 30,35 | 25,87 |
| Median | 52,8 | 51,1 |
| 75% Percentile | 75,54 | 83,96 |
| Maximum | 100 | 100 |
|  |  |  |
| Mean | 52,25 | 54,29 |
| Std. Deviation | 30,5 | 31,08 |
| Std. Error of Mean | 6,655 | 6,782 |
|  |  |  |
| Lower 95% CI of mean | 38,37 | 40,14 |
| Upper 95% CI of mean | 66,13 | 68,44 |
|  |  |  |
| Sum | 1097 | 1140 |
|  |  |  |
| D'Agostino & Pearson normality test |  |  |
| K2 | 1,18 | 2,139 |
| P value | 0,5544 | 0,3431 |
| Passed normality test (alpha=0.05)? | Yes | Yes |
| P value summary | ns | ns |
|  |  |  |
| Shapiro-Wilk normality test |  |  |
| W | 0,9578 | 0,9233 |
| P value | 0,4728 | 0,1009 |
| Passed normality test (alpha=0.05)? | Yes | Yes |
| P value summary | ns | ns |
|  |  |  |

**Table 7: D’Agostino and Shapiro-Wilk tests for Left Ventricle’s Ejection Fraction estimated by Cardiac Magnetic Resonance (CMRI)**

CMRI % EJECTION FRACTION CLOPIDOGREL TICAGRELOR

| Number of values | 21 | 21 |
| --- | --- | --- |
|  |  |  |
| Minimum | 32,27 | 36 |
| 25% Percentile | 40,27 | 47,08 |
| Median | 52,89 | 53,57 |
| 75% Percentile | 59,87 | 60,4 |
| Maximum | 78,5 | 77,81 |
|  |  |  |
| Mean | 51,94 | 54,14 |
| Std. Deviation | 12,18 | 10,37 |
| Std. Error of Mean | 2,659 | 2,264 |
|  |  |  |
| Lower 95% CI of mean | 46,39 | 49,41 |
| Upper 95% CI of mean | 57,49 | 58,86 |
|  |  |  |
| Sum | 1091 | 1137 |
|  |  |  |
| D'Agostino & Pearson normality test |  |  |
| K2 | 0,0172 | 1,138 |
| P value | 0,9914 | 0,5660 |
| Passed normality test (alpha=0.05)? | Yes | Yes |
| P value summary | ns | ns |
|  |  |  |
| Shapiro-Wilk normality test |  |  |
| W | 0,9346 | 0,981 |
| P value | 0,1700 | 0,9389 |
| Passed normality test (alpha=0.05)? | Yes | Yes |
| P value summary | ns | ns |

**Table 8: D’Agostino and Shapiro-Wilk tests for Left Ventricle’s Stroke Volume in ml estimated by Cardiac Magnetic Resonance (CMRI)**

Left Ventricle’s Stroke Volume(ml) CLOPIDOGREL TICAGRELOR

| Number of values | 21 | 21 |
| --- | --- | --- |
|  |  |  |
| Minimum | 36,29 | 48 |
| 25% Percentile | 60,57 | 65,16 |
| Median | 69,57 | 82,56 |
| 75% Percentile | 86,53 | 89,37 |
| Maximum | 101 | 124,6 |
|  |  |  |
| Mean | 70,39 | 79,71 |
| Std. Deviation | 18,99 | 19,21 |
| Std. Error of Mean | 4,144 | 4,191 |
|  |  |  |
| Lower 95% CI of mean | 61,74 | 70,96 |
| Upper 95% CI of mean | 79,03 | 88,45 |
|  |  |  |
| Sum | 1478 | 1674 |
|  |  |  |
| D'Agostino & Pearson normality test |  |  |
| K2 | 0,8402 | 0,4355 |
| P value | 0,6570 | 0,8043 |
| Passed normality test (alpha=0.05)? | Yes | Yes |
| P value summary | ns | ns |
|  |  |  |
| Shapiro-Wilk normality test |  |  |
| W | 0,9624 | 0,9499 |
| P value | 0,5660 | 0,3386 |
| Passed normality test (alpha=0.05)? | Yes | Yes |
| P value summary | ns | ns |

| Number of values | 21 | 21 |
| --- | --- | --- |
|  |  |  |
| Minimum | 10,3 | 11,63 |
| 25% Percentile | 21,06 | 26,34 |
| Median | 28,4 | 34,41 |
| 75% Percentile | 41,8 | 41,35 |
| Maximum | 74,11 | 55 |
|  |  |  |
| Mean | 34,83 | 33,85 |
| Std. Deviation | 20,46 | 11,66 |
| Std. Error of Mean | 4,464 | 2,545 |
|  |  |  |
| Lower 95% CI of mean | 25,51 | 28,54 |
| Upper 95% CI of mean | 44,14 | 39,16 |
|  |  |  |
| Sum | 731,3 | 710,9 |
|  |  |  |
| D'Agostino & Pearson normality test |  |  |
| K2 | 4,176 | 0,0838 |
| P value | 0,1239 | 0,9590 |
| Passed normality test (alpha=0.05)? | Yes | Yes |
| P value summary | ns | ns |
|  |  |  |
| Shapiro-Wilk normality test |  |  |
| W | 0,8428 | 0,9828 |
| P value | 0,0032 | 0,9596 |
| Passed normality test (alpha=0.05)? | No | Yes |
| P value summary | ** | ns |

**Table 9: D’Agostino and Shapiro-Wilk tests for Left Ventricle end systolic volume index (ml/m^2^)**

Left Ventricle end- systolic index (ml/m^2)^ CLOPIDOGREL TICAGRELOR

**Table 10: D’Agostino and Shapiro-Wilk tests for Left Ventricle End Diastolic Volume Index (ml/m^2^**

Left Ventricle End Diastolic Volume Index(ml/m^2)^ CLOPIDOGREL TICAGRELOR

| Number of values | 21 | 21 |
| --- | --- | --- |
|  |  |  |
| Minimum | 0 | 0 |
| 25% Percentile | 33,6 | 37,72 |
| Median | 53,44 | 46 |
| 75% Percentile | 65,38 | 58,86 |
| Maximum | 76,85 | 81 |
|  |  |  |
| Mean | 48,1 | 45,13 |
| Std. Deviation | 22,22 | 20,66 |
| Std. Error of Mean | 4,848 | 4,508 |
|  |  |  |
| Lower 95% CI of mean | 37,98 | 35,72 |
| Upper 95% CI of mean | 58,21 | 54,53 |
|  |  |  |
| Sum | 1010 | 947,7 |
|  |  |  |
| D'Agostino & Pearson normality test |  |  |
| K2 | 3,239 | 2,78 |
| P value | 0,1980 | 0,2491 |
| Passed normality test (alpha=0.05)? | Yes | Yes |
| P value summary | ns | ns |
|  |  |  |
| Shapiro-Wilk normality test |  |  |
| W | 0,9183 | 0,9358 |
| P value | 0,0802 | 0,1801 |
| Passed normality test (alpha=0.05)? | Yes | Yes |
|  |  |  |
| P value summary | ns | ns |

| Number of values | 21 | 21 |
| --- | --- | --- |
|  |  |  |
| Minimum | 0 | 0 |
| 25% Percentile | 33,6 | 37,72 |
| Median | 53,44 | 46 |
| 75% Percentile | 65,38 | 58,86 |
| Maximum | 76,85 | 81 |
|  |  |  |
| Mean | 48,1 | 45,13 |
| Std. Deviation | 22,22 | 20,66 |
| Std. Error of Mean | 4,848 | 4,508 |
|  |  |  |
| Lower 95% CI of mean | 37,98 | 35,72 |
| Upper 95% CI of mean | 58,21 | 54,53 |
|  |  |  |
| Sum | 1010 | 947,7 |
|  |  |  |
| D'Agostino & Pearson normality test |  |  |
| K2 | 3,239 | 2,78 |
| P value | 0,1980 | 0,2491 |
| Passed normality test (alpha=0.05)? | Yes | Yes |
| P value summary | ns | ns |
|  |  |  |
| Shapiro-Wilk normality test |  |  |
| W | 0,9183 | 0,9358 |
| P value | 0,0802 | 0,1801 |
| Passed normality test (alpha=0.05)? | Yes | Yes |
| P value summary | ns | ns |

**Table 11. D’Agostino and Shapiro-Wilk tests for myocardial infarct mean transmurality Values as percentage of Left Ventricle**

Mean transmurality values(%) CLOPIDOGREL TICAGRELOR
